# Supplementary material for: The Venom Composition of the Snake Tribe Philodryadini: ‘Omic’ Techniques Reveal Intergeneric Variability among South American Racers
Source: Toxins (Basel). 2023 Jun 27;15(7):415. doi: 10.3390/toxins15070415 (PMC10467154; doi:10.3390/toxins15070415)
Supplement: Supplementary file 1 [file toxins-15-00415-s001.zip › Supplementary_Figure_S1.pdf]

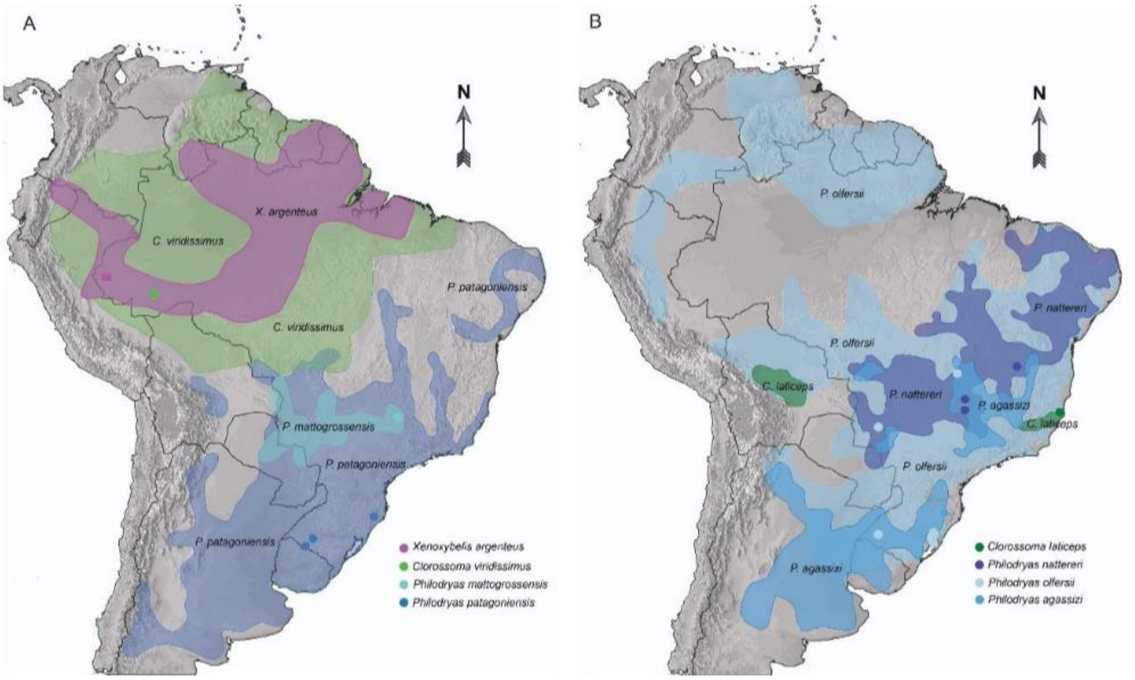

Figure S1. Geographical distribution of snakes 8 species [120] from the South American tribe Philodryadini (genus *Philodryas*, *Chlorosoma* and *Xenoxybelis*) collected between 2016 and 2021 in Brazil. Each shaded area corresponds to the distribution of each species and sampled localities are shown as dots.

Reference

120. Nogueira, C.C.; Argôlo, A.J.S.; Arzamendia, V.; Azevedo, J.A.; Barbo, F.E.; Bérnils, R.S.; Bolochio, B.E.; Borges-Martins, M.; Brasil-Godinho, M.; Braz, H.; et al. Atlas of Brazilian snakes: verified point-locality maps to mitigate the Wallacean shortfall in a megadiverse snake fauna. *South Am. J. Herpetol.* **2019**, *14*, 1–274. DOI: 10.2994/SAJH-D-19-00120.1
