# Supplementary material for: The Venom Composition of the Snake Tribe Philodryadini: ‘Omic’ Techniques Reveal Intergeneric Variability among South American Racers
Source: Toxins (Basel). 2023 Jun 27;15(7):415. doi: 10.3390/toxins15070415 (PMC10467154; doi:10.3390/toxins15070415)
Supplement: Supplementary file 1 [file toxins-15-00415-s001.zip › Supplementary_Table_S1.pdf]

Table S1. Number of reads obtained after sequencing by Illumina of the mRNA present in the Philodryadini venom glands and the number of contigs generated after *De novo* assembly.

Snake Code	Species	Merged reads	% of Merged*	Number of Contigs generated
SB0512	*Chlorosoma laticeps*	13.240.744	85.49%	204.864
SB0307	*Philodryas nattereri*	15.939.987	90.31%	263.673
SB0132	*Philodryas olfersii latirostris*	13.401.460	91.04%	162.859
SB0001	*Philodryas olfersii*	2.403.169	98.26%	57.270
SB0026	*Philodryas olfersii*	3.413.445	98.60%	82.665
SB0235	*Philodryas patagoniensis*	23.939.713	87.12%	183.351
SB0183	*Philodryas agassizii*	13.705.159	85.30%	213.881
SB0559	*Chlorosoma viridissimum*	16.716.391	84.49%	180.929
SB1590	*Xenoxybelis argenteus*	8.515.040	82.90%	171.648
SB1881	*Philodryas mattogrossensis*	7.508.732	82.41%	239.553

*The paired reads were merged based on their 3′ overlaps using PEAR version 0.9.6 (Zhang et al. 2014).
